# Metabolomic Approach for Rapid Identification of Antioxidants in *Clinacanthus nutans* Leaves with Liver Protective Potential

**DOI:** 10.3390/molecules27123650

**Published:** 2022-06-07

**Authors:** Kai Song Ng, Sheri-Ann Tan, Chui Yin Bok, Khye Er Loh, Intan Safinar Ismail, Chen Son Yue, Chui Fung Loke

**Affiliations:** 1 Department of Bioscience, Faculty of Applied Sciences, Tunku Abdul Rahman University College, Setapak, Kuala Lumpur 53300, Malaysia; ngksa-wa15@student.tarc.edu.my (K.S.N.); chuiyinbok@hotmail.com (C.Y.B.); lohke@tarc.edu.my (K.E.L.); lokecf@tarc.edu.my (C.F.L.); 2 Natural Medicine and Product Research Laboratory, Institute of Bioscience, Universiti Putra Malaysia, Serdang 43400, Malaysia; safinar@upm.edu.my; 3 Department of Physical Science, Faculty of Applied Sciences, Tunku Abdul Rahman University College, Setapak, Kuala Lumpur 53300, Malaysia; yuecs@tarc.edu.my

**Keywords:** *Clinacanthus nutans*, antioxidant, hepatoprotection, ^1^H-NMR metabolomics, partial least square analysis

## Abstract

Antioxidants are currently utilized to prevent the occurrence of liver cancer in non-alcoholic fatty liver disease (NAFLD) patients. *Clinacanthus nutans* possesses anti-oxidative and anti-inflammatory properties that could be an ideal therapy for liver problems. The objective of this study is to determine the potential antioxidative compounds from the *C. nutans* leaves (CNL) and stems (CNS). Chemical- and cell-based antioxidative assays were utilized to evaluate the bioactivities of CNS and CNL. The NMR metabolomics approach assisted in the identification of contributing phytocompounds. Based on DPPH and ABTS radical scavenging activities, CNL demonstrated stronger radical scavenging potential as compared to CNS. The leaf extract also recorded slightly higher reducing power properties. A HepG2 cell model system was used to investigate the ROS reduction potential of these extracts. It was shown that cells treated with CNL and CNS reduced innate ROS levels as compared to untreated controls. Interestingly, cells pre-treated with both extracts were also able to decrease ROS levels in cells induced with oxidative stress. CNL was again the better antioxidant. According to multivariate data analysis of the ^1^H NMR results, the main metabolites postulated to contribute to the antioxidant and hepatoprotective abilities of leaves were clinacoside B, clinacoside C and isoschaftoside, which warrants further investigation.

## 1. Introduction

Liver cancer is the sixth most frequently diagnosed cancer and the fourth major cause of cancer-related deaths worldwide, with an estimated 841,000 new cases and 782,000 deaths annually, as reported in 2018 [1]. Hepatocellular carcinoma (HCC), one of the primary liver cancers, comprises 75–85% of the cases. One of the risk factors in the development of HCC is non-alcoholic fatty liver disease (NAFLD). The progression of NAFLD to HCC is a gradual process, where oxidative stress triggers chronic inflammation that induces progressive fibrosis, leading to cirrhosis and finally cancer [2]. Oxidative stress reflects the imbalance between the generation of reactive oxygen species (ROS) and the scavenging ability of the antioxidant [3]. ROS and hydrogen peroxide are constantly produced within liver cells as byproducts of energetic metabolism, specifically in the mitochondria. An overload of free fatty acids in this organelle may lead to mitochondrial dysfunction, which affects ATP synthesis and increases ROS [4]. An excessive amount of accumulated ROS is extremely toxic to the cells and damages major cellular components, such as DNA, lipids and proteins. Furthermore, the activity of antioxidant enzymes, including glutathione peroxidase, is also impaired during mitochondrial damage, worsening the oxidative condition and, indirectly, causing liver cancer [3,4].

There is currently no specific therapy to treat NAFLD, but dietary and lifestyle modifications have shown beneficial results. The search for effective therapeutic agents to treat or prevent the initiation of NAFLD is an ongoing process. Herbal plants as well as polyherbal formulations based on Traditional Chinese Medicine, such as Danning Pian and Yiqi Sanju, have been clinically studied in NAFLD treatment, with promising results. Treatment with these herbs was reported to decrease total triglyceride and cholesterol as well as both serum alanine aminotransferase (ALT) and aspartate aminotransferase (AST) as compared to the placebo groups [5]. Furthermore, recent research by Abu-Serie and Habashy [6] reported that a polyphenolic-rich fraction of *Vitis vinifera* is able to prevent liver damage in rats due to its antioxidative and anti-necroptosis effects. In view of this, plant bioactives from *Clinacanthus nutans* could be potential natural remedies for this disease as well.

*Clinacanthus nutans* belongs to the Acantheceae family, which is native to the tropical Asian countries. It is used as an important ingredient in the traditional medicinal system of Malaysia, Thailand and Indonesia [7]. Commonly, it is known as “Sabah Snake Grass” or “Belalai Gajah” [8]. This medicinal plant has demonstrated a range of pharmacological activities, including anti-inflammatory, anti-carcinogenic and antimicrobial effects [9]. The leaves of *C. nutans* possessed antioxidant as well as anti-cancer potential towards tumor-bearing mice [10]. Both the leaves and stems of this plant exerted antimicrobial properties against *Staphylococcus aureus*, *Staphylococcus epidermis*, *Bacillus cereus* and *Escherichia coli* [11]. All these bioactivities are commonly reported in the polar and semi-polar extracts of *C. nutans,* which contain phytocompounds such as phenolics, flavonoids, sulfur-containing glucosides and phytosterols [12]. Variations of phytochemical levels are also observed in this plant species obtained from diverse geographical locations or cultivated in different environmental conditions [12,13]. In view of this, detailed information of the location where the herb is procured along with the date of harvest are provided by researchers to improve the reproducibility of results.

Metabolomics is a holistic and high-throughput analysis of highly complex mixtures for the identification of metabolites [14]. In the past decade, Nuclear Magnetic Resonance (NMR)-based metabolomics in plant analysis have evolved considerably. It has proven to be useful in fingerprinting the chemical profile of plants. The proton (^1^H) NMR has been a principal profiling tool in plant metabolomics because of its simple sample preparation, short spectral acquisition time and good reproducibility [15]. Metabolite profiling coupled with multivariate data analysis permits the identification of important metabolites without complicated isolation and purification procedures.

There is still no report on the liver protective potential of *Clinacanthus nutans*. As such, metabolomic study of the chemical compositions of *C. nutans* coupled with multivariate analyses will expedite the identification of the potential bioactive compounds responsible for both the antioxidant and liver-protective potential of this plant extract.

## 2. Results

### 2.1. Determination of Phytochemical Contents

*Clinacanthus nutans* leaf methanolic extract (CNL) was found to have higher phenolic (3.5-fold) and flavonoid (24-fold) contents than *Clinacanthus nutans* stem methanolic extract (CNS) (Table 1). Thus, CNL was predicted to possess stronger antioxidant activity due to the higher content of these secondary metabolites.

### 2.2. Antioxidant Potentials of Clinacanthus nutans Leaf and Stem Methanolic Extracts

**Figure 1:**
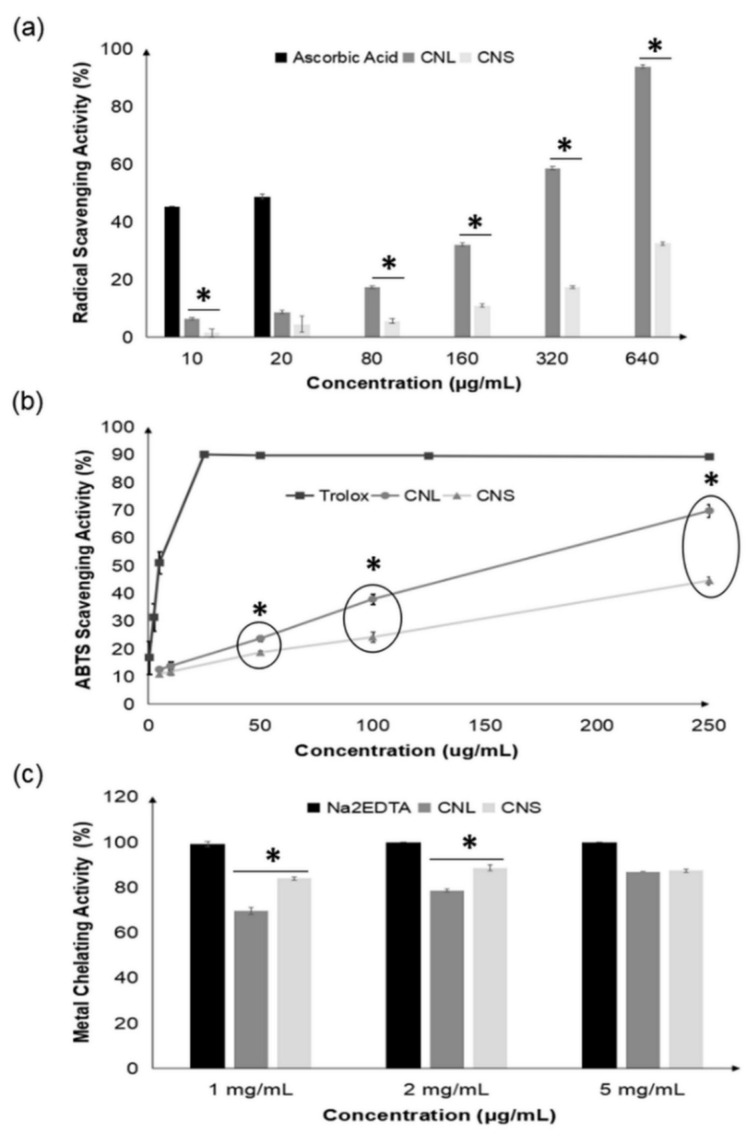
DPPH (**a**) and ABTS (**b**) radical scavenging and ferrous ion chelating (**c**) activities of CNL and CNS at various concentrations. All measurements were performed in triplicate and are expressed as mean ± standard deviation. The unpaired sample *t*-test was used to compare the variance of different datasets between CNL and CNS. Values with the symbol * are significantly different at *p* < 0.05. (CNL: *Clinacanthus nutans* leaf extract; CNS: *Clinacanthus nutans* stem extract).

Both CNL and CNS possessed significant radical scavenging activities towards DPPH and ABTS radicals, as illustrated in Figure 1a,b. CNL demonstrated higher scavenging potential towards DPPH and ABTS radicals as compared to CNS. In the DPPH radical scavenging activity assay, CNL at 640 ug/mL showed 94% scavenging activity, while CNS at the same concentration only reported 33% scavenging potential. Furthermore, CNL exhibited total inhibition of ABTS radicals (99%) at 500 µg/mL. CNS, on the other hand, scavenged only 81% of ABTS radicals at a similar concentration. Therefore, CNL was a superior radical scavenger for both radicals.

Furthermore, these two extracts were found to confer comparable abilities in reducing ferric ions as well as chelating ferrous ions. Both extracts showed high ion chelating activities, as shown in Figure 1c. At 1 mg/mL, CNS chelated ferrous ions stronger than CNL, with activity of 84%, while CNL reported a slightly lower ion chelating activity of 70%. The reducing power of CNL and CNS, on the other hand, was 12.44 ± 0.38 mg FeSO_4_/g crude extract and 11.33 ± 0.67 mg FeSO_4_/g crude extract, respectively.

### 2.3. Cytotoxic Effect of Clinacanthus nutans Leaf and Stem Methanolic Extracts on HepG2 and HaCaT Cell Lines

**Figure 2:**
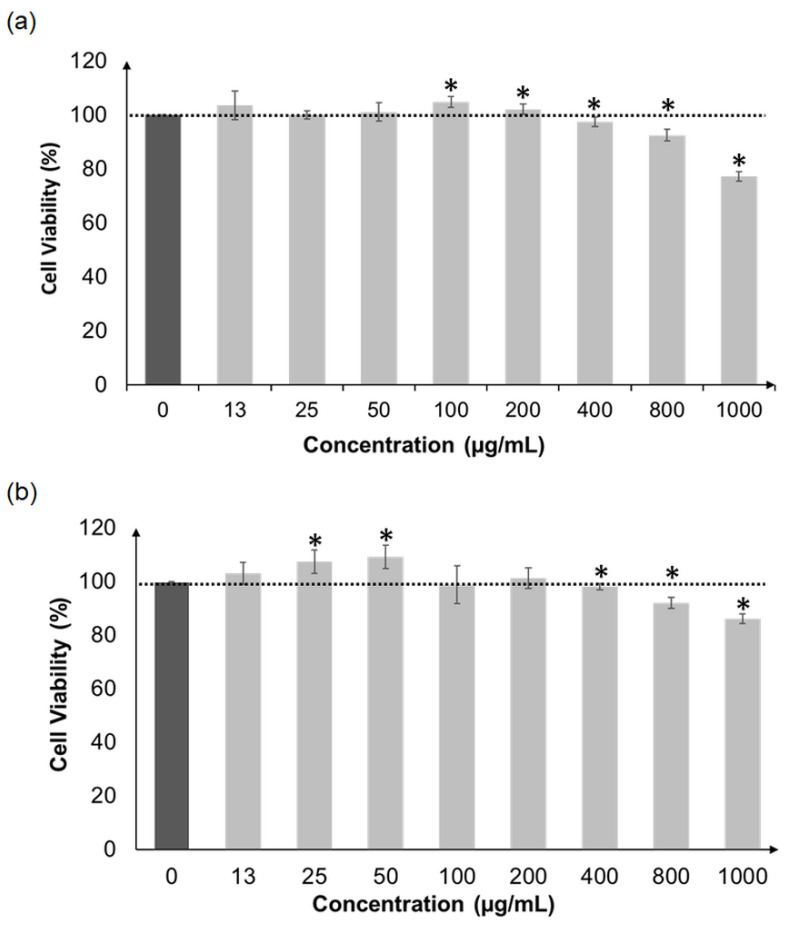
Cellular viability of HepG2 cells after pre-treatment with CNL (**a**) and CNS (**b**) at various concentrations (13 to 1000 µg/mL). All measurements were performed in triplicate and are expressed as mean ± standard deviation. Data were analyzed by comparing samples of different concentrations with untreated controls (100% viability) using the unpaired sample *t*-test. Values with the symbol * are significantly different at *p* < 0.05. (CNL: *Clinacanthus nutans* leaf extract; CNS: *Clinacanthus nutans* stem extract).

**Figure 3:**
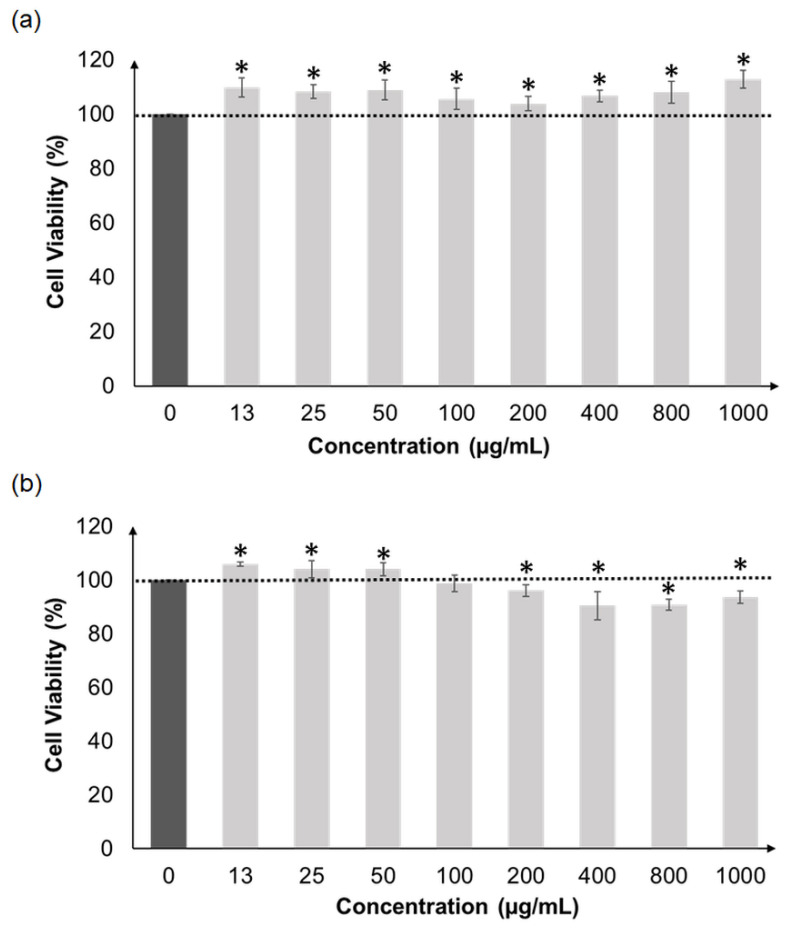
Cellular viability of HaCaT cells after pre-treatment with CNL (**a**) and CNS (**b**) at various concentrations (13 to 1000 µg/mL). All measurements were performed in triplicate and are expressed as mean ± standard deviation. Data were analyzed by comparing samples of different concentrations with untreated controls (100% viability) using the unpaired sample *t*-test. Values with the symbol * are significantly different at *p* < 0.05. (CNL: *Clinacanthus nutans* leaf extract; CNS: *Clinacanthus nutans* stem extract).

The methanolic extracts of CNS and CNL were not toxic to HepG2 liver cells when tested at a wide concentration range of 13 µg/mL to 1000 µg/mL (Figure 2). A similar observation was made when these extracts were treated on normal skin cells (HaCaT), since viabilities remained high (Figure 3). Overall, both extracts were concluded to be non-toxic, and concentrations of 13, 25 and 50 µg/mL were selected for subsequent analyses.

### 2.4. Direct and Protective Effects of Clinacanthus nutans Extracts on Intracellular Reactive Oxygen Species (ROS) Generation of HepG2 Cell Line

**Figure 4:**
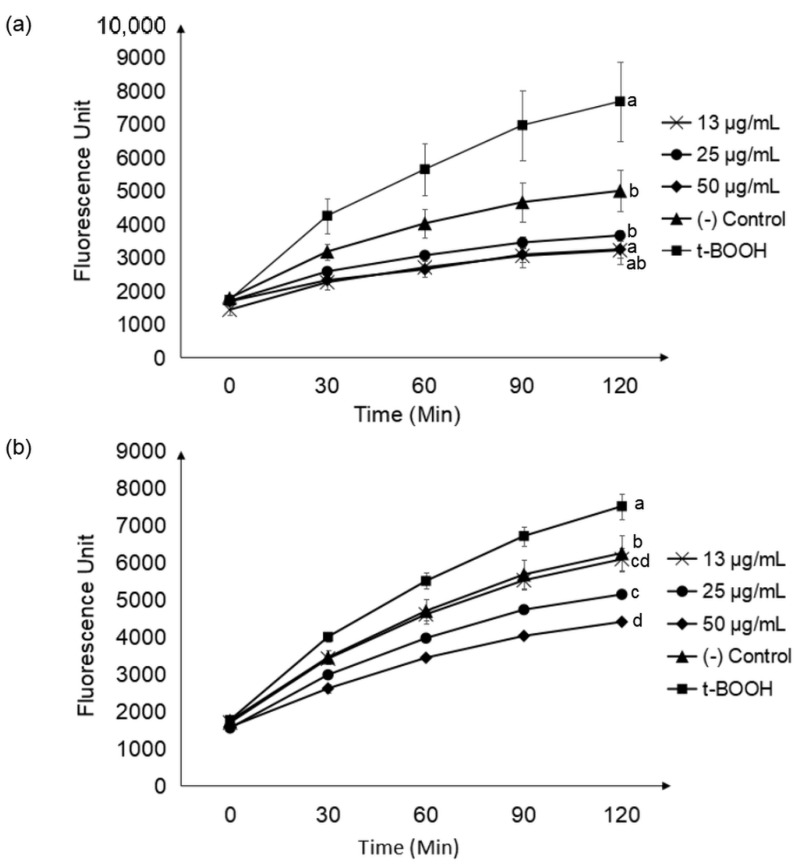
Reduction of innate ROS level after treatment with CNL (**a**) and CNS (**b**) in HepG2 liver cells at various concentrations. ROS production was monitored for 2 h using DCFH-DA assay. All measurements were performed in triplicate and are expressed as mean ± standard deviation. One-way ANOVA was used to compare the variance between different concentrations of samples at 120 min. Means with a common alphabet are significantly similar at *p* < 0.05. (CNL: *Clinacanthus nutans* leaf extract; CNS: *Clinacanthus nutans* stem extract).

The effects of CNL and CNS extracts towards the innate reactive oxygen species (ROS) levels were examined using the concentrations 13, 25 and 50 µg/mL. It was found that both extracts at all tested concentrations were able to decrease innate ROS as compared to untreated control cells. The positive control, tert-butyl hydroperoxide (t-BOOH), increased ROS exponentially as expected, indicating the cell model system was working (Figure 4).

The protective effects of CNL and CNS towards oxidative stress-induced HepG2 cells were determined as well in this research. Based on Table 2, ROS production after induction with t-BOOH in HepG2 cells was reduced after pre-treatment with both extracts. Results indicated that cells pre-treated with CNL and CNS managed to significantly reduce the ROS level by 17.44% and 9.77%, respectively, at a concentration of 50 µg/mL. At a lower concentration of 13 µg/mL, CNL also demonstrated more effective ROS reducing potential by lowering ROS by 7.14%, while CNS decreased ROS by only 1.74%.

### 2.5. ^1^H-NMR Spectra of Plant Extracts and Metabolite Identification

**Figure 5:**
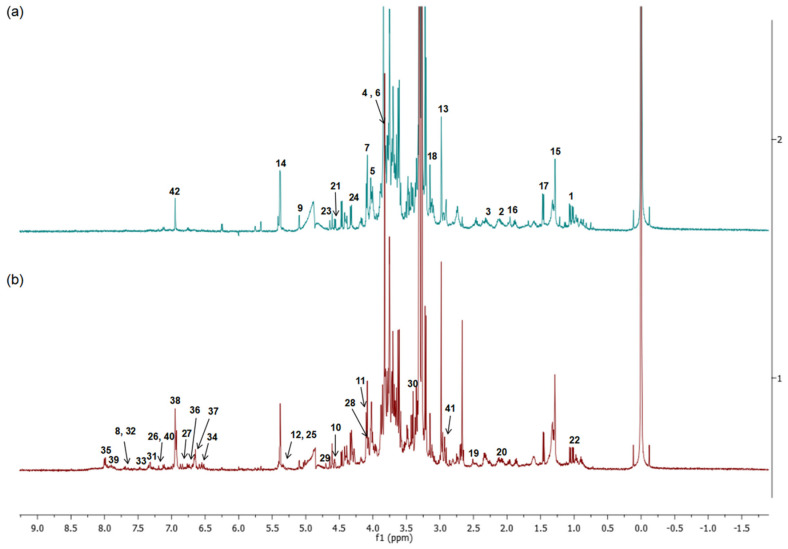
Representative ^1^H NMR spectra (−2 to 9.0 ppm) of *Clinacanthus nutans* leaf (**a**) and stem (**b**) extracts. Values on the *X*-axis are the chemical shift (ppm) relative to TMS at 0.00 ppm.

Figure 5 shows the ^1^H NMR spectra for each sample extract. Visual inspection of the aligned spectra determined the metabolite intensity, which differed due to the different parts of *C. nutans* used, specifically, the aliphatic and aromatic groups in the region of 6.0–8.5 ppm. The spectral resonances were assigned based on previous works, including those by Khoo et al. [16], Khoo et al. [17], and the library of Chenomx NMR suite 5.1 professional (Chenomx Inc., Edmonton, AB, Canada) by the peak fitting method, as tabulated in Table 2. In order to obtain a better understanding of the metabolite variations, the chemometric dataset was analyzed using multivariate data analysis by SIMCA P.14.1.0 software (Umetrics, Umeå, Sweden).

The identification of metabolites using ^1^H-NMR spectra is shown in Table 3. Fifteen of the compounds had been previously reported as constituents of *C. nutans*. These included lipids (monoacylmonogalactosylglycerol), sulfur-containing glucosides (clinacoside A, clinacoside B, clinacoside C, cycloclinacoside A1, and cycloclinacoside A2), flavones (isoorientin, isovitexin, isoschaftoside, orientin, schaftoside, and vitexin), and terpenoids (lupeol, stigmasterol, and β-sitosterol). Other compounds were identified based on a comparison with the literature data and the Chenomx database. They were listed as amino acids (alanine, glutamate, glutamine, proline and valine), fatty acid, acetic acid, lactic acid, succinic acid, malonic acid, pimelic acid, citric acid (an organic acid), carbohydrates (fructose, sucrose, α-glucose and β-glucose), ascorbic acid, choline, anthranilate, flavanol (catechin and epigallocatechin), aromatic acids (gallic acid and 3-O-methylgallic acid) and adenine.

**Figure 6:**
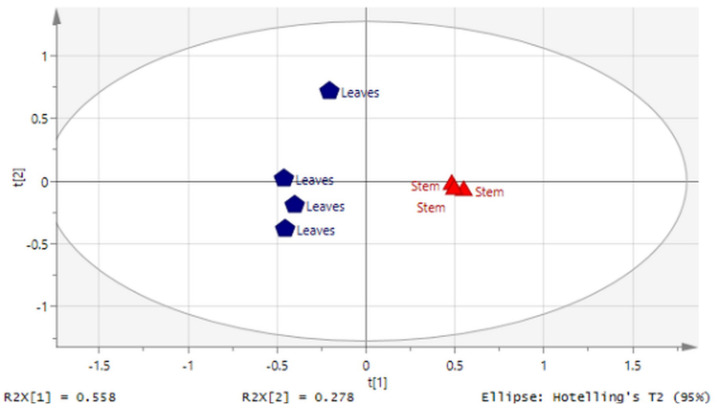
The respective principal component analysis (PCA) score plots of ^1^H NMR data representing *Clinacanthus nutans* samples.

The influence of different parts of *C. nutans* on metabolite contents present in the extracts were further analyzed using principal component analysis (PCA). The PCA score plot, constructed from the ^1^H NMR spectral data, illustrated the similarities and differences among samples based on the clustering features of samples (Figure 6). The cross-validation of the constructed PLS model was proven acceptable based on goodness of fit (R2) and predictive power (Q2) of R2X = 0.836 and Q2 = 0.49. In Figure 6**,** the PCA score plot showed that CNL and CNS were separated by PC1. CNL was clustered on the lower left quadrant, while CNS was clustered on lower right quadrant.

### 2.6. Relationship between Bioactivities and Plant Metabolites Using Partial Least-Square Analysis (PLS)

**Figure 7:**
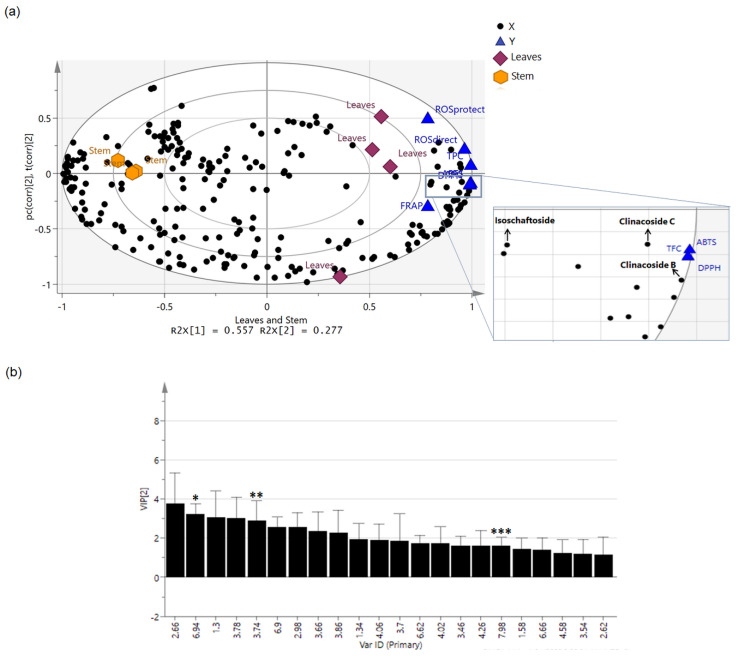
The partial least square (PLS) biplot (**a**) showing the correlation of the metabolites with chemical and cell-based assay results in CNL and CNS. Metabolites with variable importance in the projection (VIP) value ≥ 1.0 are labelled in black (**b**). The VIP metabolites with the respective symbols (*, Clinacoside B; **, Clinacoside C; ***, Isoschaftoside) are identified as metabolites contributing to the bioactivities.

In order to further investigate the relationship between the metabolites and the hepatoprotective activities, partial least square (PLS) was used. PLS is a supervised analysis that provides dual techniques in which it connects the information obtained from two blocks of data matrices and displays the correlation in a single graphical plot, as depicted in Figure 7a. In this study, X independent variables represented the ^1^H-NMR spectra data obtained from plant samples, while the Y respondent variables represented the chemical and cell-based assay results produced by the extracts. The cross-validation of the constructed PLS model was proven acceptable based on the goodness of fit (R2) and predictive power (Q2) of R2Y = 0.986 and Q2 = 0.946. In Figure 7b, the VIP values of ≥1.0 led to the selection of the compounds responsible for the bioactivities based on the listed ^1^H-NMR chemical shifts in Table 3.

## 3. Discussion

Phenolic compounds are one of the major bioactive molecules widely detected in plants. These bioactive compounds are efficient antioxidants, as they possess the ability to donate electrons to free radicals, thus breaking the chain reaction of oxidation [18]. In addition, they are also able to chelate metal ions and regulate the production of endogenous antioxidants such as glutathione [19,20]. The herbal plant *Clinacanthus nutans* was reported to contain phenolic compounds that contribute to its vast bioactivities, such as anti-inflammatory, anti-cancer, anti-venom and anti-diabetic properties [21]. A number of flavonoids, such as *C*-glycosylated flavone, flavone, flavonol, flavanone and isoflavone were isolated from the leaves of *C. nutans* [22]. Furthermore, phenolic acids including caffeic acid, chlorogenic acid, ferulic acid, protocatechuic acid and cinnamic acid were also identified in this plant [23]. Other than phenolic compounds, *C. nutans* contained triterpenoids, phytosterols, lipids and sulfur-containing compounds [12]. These compounds showed antioxidative activities as well [24].

Based on Table 1, the leaf extract of *C. nutans* (CNL) contained higher phenolic and flavonoid contents as compared to the stem extract (CNS) using a similar method of extraction, which was maceration in methanol. Maceration was the method of choice, as it enabled extraction of thermolabile compounds as compared to the Soxhlet technique [25]. Greener extraction protocols that do not require organic solvents are also currently available, such as the supercritical carbon dioxide extraction method. Nevertheless, this technique was found to be ineffective in extracting polar compounds from *C. nutans,* such as polyphenols, due to the non-polar characteristics of carbon dioxide [22]. Furthermore, supercritical CO_2_ extraction is costly to perform and requires highly trained personnel to operate the system. Maceration, on the other hand, is economical and the easiest conventional method to execute. Importantly, macerated *C. nutans* leaves in methanol had successfully extracted various phenolic compounds, flavonoids, diterpenes, saponins and phytosterols in a previous study [26]. Hence, this extraction technique was used in this investigation.

Chemical-based antioxidative assays were performed to determine the antioxidant efficacy of CNL and CNS. Earlier, researchers had tested the methanolic extract of *C. nutans* for both DPPH and ABTS free radical scavenging activities, and the results were promising [27]. The *C. nutans* extract demonstrated the ability to scavenge DPPH and ABTS free radicals at the IC_50_ values of 1.18 ± 0.05 mg/mL and 0.98 ± 0.05 mg/mL, respectively. Similarly, in our work, both leaves and stems of this plant exhibited strong radical scavenging potentials. Nevertheless, CNL, which contained higher phenolic and flavonoid contents, was the better scavenger as compared to CNS. This could be due to the fact that the abundance of phenolics and flavonoids present in the leaves was able to act directly on the oxidative agents by donating hydrogen atoms or electrons to the free radicals, therefore stabilizing the radicals. Ultimately, this would prevent the chain reaction of oxidative stress, thus alleviating oxidative damage [28,29].

Our findings indicated that *C. nutans* was also able to reduce ferric ions to ferrous ions in the ferric-reducing antioxidant power assay (FRAP), as well as possessing the ability to chelate ferrous ions. The phenolics, tannins and alkaloids contents of *C. nutans* leaves were recently found to contribute towards these bioactivities [30]. Most reported antioxidants are naturally well-known chelating agents, as they possess the binding affinity to most positively charged metal ions, thus reducing metal toxicity [31]. Heavy metals are one source of environmental pollution, which contributes to long-term negative health effects. Many heavy metals are able to perform oxidation-reduction (redox) activities, giving rise to the generation and overproduction of reactive oxygen species (ROS) and leading to oxidative damage [32].

In order to proceed to cell-based antioxidant activities, the cytotoxicities of CNL and CNS were first examined using the HepG2 liver cell line, which was the model system used for the assays. Both extracts did not cause any form of cell death from concentrations 1000 µg/mL and below. In addition, these extracts were also non-toxic towards normal human skin cells, HaCaT, at the same concentration range. This was supported by Vajrabhaya and Korsuwannawong [33], who showed that *C. nutans* did not display any cell toxicity towards normal human gingival fibroblast cells. In another study, ethyl acetate: hexane fractions of *C. nutans* were found to be non-toxic towards normal fibroblast NIH 3T3 and L929 normal connective cells [34]. Insignificant cytotoxicity of the methanolic extract of *C. nutans* towards Saos-2 osteosarcoma cells was reported as well [35]. Therefore, extract concentrations from 13 to 50 µg/mL were deemed safe to be used in subsequent cell-based assays.

DCFH-DA assay is based on the principle that 2′,7′-dichlorodihydrofluorescein diacetate (DCFH-DA) is taken up by the cells, where cellular esterase cleaves off the acetyl group of DCFH-DA, which results in the production of DCFH. ROS presence in the cells oxidizes DCFH to DCF, which emits green fluorescence at an excitation wavelength of 485 nm and an emission wavelength of 530 nm [36]. CNL and CNS were initially treated on the cells to investigate whether both extracts were capable of reducing the innate ROS level. In normal conditions, the level of cellular ROS is stable in a dynamic equilibrium, and this balance is modulated by cellular processes that generate ROS and eliminate them. The main source that generates cellular ROS is the mitochondrial oxidative metabolism, which releases ROS as a byproduct [37]. After treatment with CNL and CNS (at 50 µg/mL), it was found that the innate ROS was reduced by 35% and 30%, respectively.

In this study, we also investigated the protective effects of CNL and CNS treatment to overcome induced oxidative stress. The pro-oxidant utilized in this work was tert-butyl hydroperoxide (t-BOOH), a membrane-permeant oxidant that has been widely used in the evaluation of mechanisms of cellular alterations resulting from oxidative stress in cells and tissues [38,39]. Treatment with t-BOOH leads to depletion of GSH, lipoperoxidation and the onset of mitochondrial permeability transition (MPT), causing cell damage and death [38,40]. Furthermore, HepG2 treated with this pro-oxidant exhibited high ROS production in cells, thus validating its use in this experiment [41,42]. Interestingly, both plant extracts displayed reduced cellular ROS production in the range of 1.74% to 17.44%. The leaf extract showed superior ROS reduction potential in comparison to the stem extract. Previous research demonstrated similar ROS reduction potential towards t-BOOH-induced cellular stress using various plant materials, such as *Phyllanthus phillyreifolius* [43], olive pomace [41] and cocoa phenolic [44] extracts. These free radicals were reduced or inhibited by phytocompounds, especially phenolics, due to the transfer of a hydrogen atom from its hydroxyl group to the radicals [45].

A number of research groups have reported that the leaves of *Clinacanthus nutans* are indeed stronger antioxidants as compared to stems, coinciding with our results. It has been reported that the alcoholic leaf extract of this plant possesses higher total phenolic contents and radical scavenging activities as compared to the stem extract, regardless of the extraction procedures (sonication and maceration) [17] and solvents (hexane, methanol and water) [23] utilized. Importantly, this extract was able to alleviate induced-oxidative stress in a murine model system by upregulating the expression of antioxidant enzymes in liver cells, possibly due to its high phenolic composition [23]. The methanolic leaf extract also demonstrated anti-inflammatory potential in vivo, based on the reduction of nitric oxide levels in the blood [10]. Researchers postulated that, in addition to phenolic compounds, other phytoconstituents such as sulfur-containing compounds, which include the clinamides and clinacosides present in *C. nutans* leaves, could be responsible for the bioactivities either alone or through synergistic action [16,24].

In fact, there are a number of phytocompounds identified in *C. nutans,* such as lupeol, stigmasterol, β-sitosterol, betulin and myricyl alcohol [46]. Other than this, there are six known C-glycosyl flavones, including orientin, isoorientin, isomollupentin 7-O-β-D-glucopyranoside, schaftoside, isovitexin and vitexin, that were isolated from the butanol and water extracts of this plant [47]. Sulfur-containing glucosides, such as clinacoside A, clinacoside B, clinacoside C and cycloclinacoside A1, were also detected in the butanol fraction [48].

In the last few years, metabolomics has been increasingly used in herbal medicine for various applications, such as determining the optimum conditions for cultivation, detecting adulterants, evaluating biological efficacies and assessing qualities. This is because NMR results are easily reproducible, and the sample needed for preparation is minimal. In addition, it can be used to identify both primary and secondary metabolites simultaneously without the need for any kind of fractionation or separation process [49]. Therefore, proton NMR spectroscopy was chosen for this study to identify the potential metabolites responsible for the bioactivities in CNL.

NMR data from CNL were separated from the CNS by PC1. Figure 6 suggests that the separation of leaf samples from stem samples by PC1 may be due to the presence of phenolic compounds and sulfur-containing glucosides. These results are further supported by the information provided in Table 3. According to Table 3, the comparison between the ^1^H NMR spectra of the leaves and stem samples showed marked differences, especially in the aromatic region of δH 6.00–8.00. This suggested that the leaf extract may contain more aromatic compounds [50]. Moreover, the stem extract was found to contain more sugars and carbohydrate-types of compounds when compared with the leaves.

PLS regression analysis with cross-validation was applied to further confirm the correlations between the biological activities and the chemical constituents present in the *C. nutans* extracts, presented in the form of a biplot (a combination of score and loading plots). In Figure 7a, the PLS biplot showed separated clusters. PC1 separated the leaf extract from the stem extract. The leaf extract was strongly correlated with total phenolic and flavonoid contents, ABTS and DPPH radical scavenging activities, ferric reducing antioxidant power potential, as well as ROS direct and protection assays. On the other hand, the stem extract was located in the opposite and negative site of the plot, showing correlation to metal chelating activity only.

The chemical constituents that contributed to the separation between leaf extracts and stem extracts included carbohydrates (sucrose, fructose and β-glucose), primary metabolites (anthranilate, adenine, proline, asparagine, acetic acid and succinic acid) and secondary metabolites (phenolic compounds, terpenoids and sulfur-containing glucosides). Based on Table 3, leaf extracts were found to contain a higher number of phytochemicals compared to stem extracts, which could be the main contributor of its stronger bioactivities. The main phytocompounds present in CNL postulated to confer the beneficial effects were isoschaftoside, clinacoside B and clinacoside C, as the chemical shifts of these compounds were detected to be at close proximity to the Y-variables in the PLS biplot. Moreover, the compounds were further determined by using variable importance in the projection (VIP), which was used to calculate the influence of the X-variables on the PLS model. In the VIP plot, isoschaftoside, clinacoside B and clinacoside C were identified to have VIP values of more than 1.0. This result gave a strong indication of the possible contribution of these three compounds towards the observed bioactivities.

Isoschaftoside and clinacoside B as well as clinacoside C are known to be strong antioxidants, while the former was also reported to exert hepatoprotective abilities [9]. Isoschaftoside, a major component of *Abrus mollis* extracts, exhibited hepatoprotective potential against carbon tetrachloride (CCL_4_)-induced hepatic fibrosis in a rat model [51]. The other two phytocompounds, clinacoside B and C, have been mentioned to be effective anti-inflammatory agents. Both clinacosides showed anti-neuroinflammatory effects against lipopolysaccharide (LPS)-induced neurotoxicity in the in vivo rat model [9]. Furthermore, they displayed inhibitory effects towards human neutrophil elastase enzymes in silico, which indirectly signified their abilities to prevent inflammation in the respiratory system [13,52]. Based on their proven antioxidative and anti-inflammatory potentials, these three compounds are indeed promising hepatoprotective agents that warrant further investigations. Among the three compounds, isoschaftoside is the most stable phytochemical detected in *C. nutans* from different geographical locations, regardless of the environmental conditions [13]. Thus, it is deemed a suitable chemical marker to standardize *C. nutans* extract or even raw materials.

At the present moment, the *Clinacanthus nutans* leaf extract is undergoing bioactivity guided fractionation to further confirm the relevance of isoschaftoside, clinacoside B and clinacoside C towards the observed effects on liver cells. The mechanistic action of the active fraction will eventually be determined, including its influence on vital antioxidant pathways such as Keap1-Nrf2, which has a significant impact on liver health.

## 4. Materials and Methods

### 4.1. Chemicals and Reagents

Absolute and deuterated methanol, tetramethylsilane (TMS), ascorbic acid, Folin-Ciocalteu’s phenol reagent and gallic acid were procured from Merck (Darmstadt, Germany). 2,2-Diphenyl-1-picrylhydrazyl (DPPH) reagent, 2,2′-azino-bis (3-ethylbenzthiazoline-6-sulfonic acid) diammonium salt (ABTS) reagent, 3-(2-pyridyl)-5,6-diphenyl-1,2,4-triazine-p, p’-disulfonic acid monosodium salt hydrate (Ferrozine), (±)-6-hydroxy-2,5,7,8-tetramethylchromane-2-carboxylic acid (Trolox) and quercetin were obtained from Sigma-Aldrich (St. Louis, MO, USA). Cell culture chemicals and media were purchased from Thermo Fisher Scientific, Inc. (Waltham, MA, USA).

### 4.2. Preparation of Clinacanthus nutans Leaf (CNL) and Stem Extracts (CNS)

*Clinacanthus nutans* was purchased from TKC Herbal Nursery Sdn. Bhd. in Jelebu, Negeri Sembilan (Malaysia), in April 2018. Authentication of the plant specimen was made and deposited at the herbarium of the Biodiversity Unit, University Putra Malaysia (UPM), Serdang, Selangor, Malaysia. The voucher number given was SK3266/17. Leaf and stem samples were washed thoroughly using distilled water, then dried in an oven at 40 °C and ground into fine powder. Powdered samples were then macerated in 80% methanol (*v*/*v*) at the ratio of 1:10 (*w*/*v*) in the dark. Next, the extract solvent was filtered using Whatman filter paper No. 1 in a Buchner funnel. The filtrates were then concentrated by a rotary evaporator before freeze-drying. The freeze-dried extracts were kept in a −20 °C freezer for subsequent experiments.

### 4.3. Determination of Phytochemical Contents

#### 4.3.1. Total Phenolic Content

Total phenolic contents of the extracts were quantitated using the Folin-Ciocalteu method as described by Stanković et al. [53] with slight modifications. The extracts (0.5 mL) were mixed with 2.5 mL of 10% Folin-Ciocalteu’s reagent and then incubated in the dark for 8 min. The mixtures were then added with 7.5% sodium carbonate and incubated in darkness again for 30 min in a 40 °C water bath. The absorbance readings were measured by using a UV-Vis spectrophotometer (Hitachi U-2900, Tokyo, Japan) at 765 nm. Total phenolic contents of the extracts were expressed as gallic acid equivalents (mg GA/g crude extract).

#### 4.3.2. Total Flavonoid Content

Total flavonoid contents of the extracts were quantitated using the aluminum chloride colorimetric method as described by Stanković et al. [53] with slight modifications. The extracts (3 mL) were added with 1.5 mL of 2% aluminum chloride, followed by 120 mM potassium acetate. The mixtures were left in the dark for 30 min. The absorbance readings were measured using a UV-Vis spectrophotometer (Hitachi U-2900, Tokyo, Japan) at 415 nm. Total flavonoid contents of the extracts were expressed as quercetin equivalent (mg QE/g crude extract).

### 4.4. Chemical-Based Antioxidant Assays

#### 4.4.1. DPPH Radical Scavenging Activity Assay

DPPH radical scavenging activities of the extracts were determined according to the method published by Shen et al. [54] with slight modifications. The extracts (3 mL) were added with 1 mL of 0.1 mM DPPH reagent and then left in dark at room temperature for 30 min. Absorbance readings were measured by using a UV-Vis spectrophotometer (Hitachi U-2900, Tokyo, Japan) at 517 nm after 30 min.

#### 4.4.2. ABTS Radical Scavenging Activity Assay

ABTS radical scavenging activities of the extracts were determined based on the method published by Lee et al. [55]. ABTS working reagent was prepared by mixing 7 mM ABTS and 140 mM potassium persulfate and then incubated in dark at room temperature for 16 h. The working reagent was diluted with absolute methanol at the ratio of 1:44 before use. Diluted ABTS working reagent was added to the extracts in a 96-well microplate and kept in darkness for 6 min at room temperature. Absorbance readings were measured by using a microplate reader (TECAN, Groding, Austria) at 734 nm after 6 min.

#### 4.4.3. Ferric Ion Chelating Activity Assay

Ferrous ion chelating activity of the extracts was determined by using the method from Chew et al. [56]. The extracts (1 mL) were added with equal volume of 0.1 mM ferrous sulphate, followed by 1 mL of 0.25 mM Ferrozine. The mixtures were then incubated in the dark at room temperature for 10 min. Absorbances were recorded at 562 nm using a UV-Vis spectrophotometer (Hitachi U-2900, Tokyo, Japan).

#### 4.4.4. Ferric Reducing Antioxidant Power (FRAP) Assay

Ferric reducing antioxidant power of the extracts was determined according to the method by Benzie and Strain [57]. The FRAP working reagent was prepared by mixing 300 mM acetate buffer (pH 3.6), 10 mM TPTZ in 40 mM hydrochloric acid, and 20 mM iron chloride at the ratio of 10:1:1. The reagent was warmed in a 37 °C water bath before use. The extracts were then added with the FRAP working reagent and incubated at room temperature for 30 min. Absorbance readings were measured at 593 nm using a UV-Vis spectrophotometer (Hitachi U-2900, Tokyo, Japan) and then expressed in mg FeSO_4_/g crude extract.

### 4.5. Cell-Based Antioxidant Assays

#### 4.5.1. Cytoxicity Assays

The cells were seeded (1 × 10^4^ cells/well in 96-well plates) and cultured in a 37 °C CO_2_ incubator for 48 h. After that, the cells were washed twice using 1× PBS before treatment with the *C. nutans* extracts (13 to 1000 µg/mL) for 24 h at 37 °C in a 5% CO_2_ incubator. The control cells were added with 0.1% DMSO, which was the solvent used to dissolve the extracts. After 24 h, the culture medium was aspirated, and cells were washed with 1× PBS twice, followed by addition of 100 µL of fresh DMEM F-12 with 10% FBS into each well. Subsequently, 10 μL of MTT solution was added to each well, and the plate was incubated for 2.5 h at 37 °C in a 5% CO_2_ incubator. After the formazan crystal had formed, 100 μL of 10% SDS with 0.01M of HCl were added into each well. After an overnight incubation, the intensity of dissolved formazan crystals was quantified using a microplate reader at 540 nm absorbance. The results were expressed as percentage (%) viability [58].

#### 4.5.2. ROS Direct

The intracellular ROS production was assessed using the method published by Wang and Joseph [59]. The cells were seeded in 24-multiwell plates (2 × 10^5^ cells/well) with FBS-containing medium. The medium was changed to a serum-free medium before the experiment commenced. After one day, 10 µL DCFH (5 µM) was added to the wells and incubated for 45 min before addition of extracts at various concentrations (13 to 50 µg/mL). Multiwell plates were immediately measured with a fluorescence microplate reader (λex = 485 nm, λem = 530 nm). ROS production was measured every 30 min for 2 h.

#### 4.5.3. ROS Protection

The cells were seeded in 24-multiwell plates (2 × 10^5^ cells/well) with FBS-containing medium. After the medium was removed, cells were treated with different concentrations of extracts (13 and 50 µg/mL) for 20 h. A volume of 10 µL DCFH (5 µM) was added to the wells, incubated and washed prior to the addition of 0.5 mL of 400 µM t-BOOH, the prooxidant, to all wells except the control. Multiwell plates were immediately measured with a fluorescence microplate reader (λex = 485 nm, λem = 530 nm). ROS production was measured every 30 min for 2 h [59].

### 4.6. ^1^H NMR-Based Metabolomic Study

The plant extracts were subjected to ^1^H-NMR analysis according to the procedure reported by Khoo et al., using a 500 MHz Varian INOVA NMR spectrometer (Varian Inc., Palo Alto, CA, USA) [17]. First, each sample was weighed (5 mg) and dissolved in 1 mL of deuterated methanol with 0.3% of tetramethylsilane (TMS). The samples were sonicated for 20 min at room temperature and centrifuged at 13,000 rpm for 10 min. Then, 0.6 mL of supernatant was transferred to a NMR tube to perform NMR analysis using a pre-saturation (PRESAT) setting. After that, the acquired NMR spectra underwent phasing and baseline correction using Chenomx software Version 5.1 (Edmonton, AB, Canada). Water region (δH 4.8–5.05) and residual methanol (δH 3.23–3.34) were excluded. Spectral intensities were binned by equal width (δH 0.04), corresponding to the region of δH 0.50–10.00. The processed and bucketed data were then subjected to multivariate data analysis (MVA) using SIMCA-P+ Version 14.1 (Umetrics AB, Umeå, Sweden). Data were mean-centered and Pareto-scaled before proceeding to Principal Component Analysis (PCA) and Partial Least Squares (PLS) regression analysis. Data were visualized using biplot analysis with the scores plot of the two principal components (PC1 and PC2), in which each point represented an individual spectrum of a sample. Variable Importance in Projections (VIP) was carried out to further confirm the significance and validity of the PLS model. The data were compared against the Chenomx library Version 5.1 (Edmonton, AB, Canada), Human Metabolome Database (HMDB) and previously reported data for compound identification.

### 4.7. Statistical Analysis

Results obtained from this study were analyzed using IBM SPSS version 21.0. Data were first tested for homogeneity of variances by the Levene test. For multiple comparison, one-way ANOVA was carried out, followed by the Tukey test (when variances are homogenous) or Tamhane test (when variances are not homogenous). In order to compare only two sample groups, the unpaired sample *t*-test was employed. All data were examined at the level of significance where *p* < 0.05.

## 5. Conclusions

The *Clinacanthus nutans* leaf extract, rich in phenolics and flavonoids, displayed stronger antioxidative activities in both chemical- and cell-based assays as compared to the stem extract. NMR metabolomic analyses deduced that the presence of isoschaftoside, clinacoside B and clinacoside C in the leaves could potentially contribute to the observed bioactivities. Nevertheless, more in-depth investigations should be conducted to validate these findings. In addition to in vitro experiments, in vivo studies using murine models of NAFLD could provide a more detailed perspective on the efficacy of the proposed therapeutics. This is primarily due to the intricacy of the living systems that perform various metabolic and physiologic functions that could influence medication response.

## Figures and Tables

**Table 1:** Total phenolic content (TPC) and total flavonoid content (TFC) of *Clinacanthus nutans* leaf (CNL) and stem (CNS) methanolic extracts.

Extract	Average TPC (mg GA/g Crude Extract)	Average TFC (mg QE/g Crude Extract)
**CNL**	21.75 ± 2.41 ^a^	90.17 ± 0.58 ^a^
**CNS**	6.00 ± 0.43 ^b^	3.82 ± 0.25 ^b^

All measurements were performed in triplicate and the values are expressed as the mean ± standard deviation. Means with different superscript letters are significantly different (*p* < 0.05) when comparing CNL and CNS for each phytochemical content using the unpaired sample *t*-test.

**Table 2:** Reduction of ROS level (%) in oxidative stress-induced HepG2 cells after pre-treatment with CNL and CNS as compared to untreated controls using DCFH-DA assay.

Concentration	ROS Reduction (%)	
	CNL	CNS
**13 µg/mL**	7.14 ± 4.163 ^a^	1.74 ± 2.292 ^b^
**50 µg/mL**	17.44 ± 3.200 ^a^	9.77 ± 5.485 ^b^

All measurements were performed in triplicate and are expressed as mean ± standard deviation. Different samples at similar concentrations were compared using unpaired sample *t*-test. Means with different superscript letters are significantly different (*p* < 0.05). (CNL: *Clinacanthus nutans* leaf extract; CNS: *Clinacanthus nutans* stem extract).

**Table 3:** ^1^H NMR characteristic signals of identified metabolites in *Clinacanthus nutans* extracts.

Metabolites	^1^H-NMR Characteristic Signals	
	CNL	CNS
**Primary and intermediate metabolites**		
(1) Valine ^a^	3.60 (d, J = 11.1 Hz), 2.265 (m), 1.02 (d, J = 6.9 Hz)	3.61 (d, J = 8.0 Hz), 2.28 (m), 1.02 (d, J = 6.9 Hz)
(2) Glutamine ^a^	7.60 (m), 6.88 (s), 3.77 (m), 2.45 (m), 2.11 (m)	7.60 (m), 6.88 (s), 3.77 (m), 2.45 (m), 2.11 (m)
(3) Glutamate ^a^	3.75 (q), 2.35 (m), 2.08 (m)	3.75 (m), 2.35 (m), 2.08 (m)
(4) Alanine ^a^	3.89 (q), 1.45 (d, J = 7.2 Hz)	3.78 (q), 1.46 (d, J = 7.1 Hz)
(5) Choline ^a^	4.08 (m), 3.47 (m), 3.19 (s)	4.08 (m), 3.47 (m), 3.2 (s)
(6) Betaine ^a^	3.87 (s), 3.26 (s)	3.87 (s), 3.26 (s)
(7) Proline ^a^	—	4.12 (t), 3.41 (dt), 3.33 (dt), 2.34 (sext), 2.04 (m), 1.95 (m)
(8) Anthranilate ^a^	7.70 (m), 7.29 (t), 6.94 (d, J = 10.9 Hz), 6.86 (t)	—
(9) *α*-Glucose ^b^	5.10 (d, J = 3.7 Hz)	5.10 (d, J = 3.9 Hz)
(10) *β*-Glucose ^b^	—	4.56 (d, J = 7.9 Hz)
(11) Fructose ^b^	—	4.17 (d, J = 7.6 Hz)
(12) Sucrose ^b^	5.39 (d, J = 3.8 Hz)	—
(13) Asparagine ^b^	—	2.95 (m), 2.77 (m)
(14) Monoacylmonogalactosyl glycerol ^b^	5.39 (m), 4.16 (m), 4.01 (m), 3.75 (m), 3.70 (m), 3.52 (m), 2.81 (s), 2.10 (d, J = 18.8 Hz), 2.06 (m), 1.30 (m), 1.29 (s)	5.36 (m), 4.17 (d, J = 7.6 Hz), 4.02 (m), 3.75 (m), 3.7 (m), 3.47 (m), 3.51 (m), 2.81 (m), 2.11 (m), 2.06 (m), 1.30 (m), 1.28 (s)
(15) Fatty acid ^b^	1.31 (m)	1.36 (m)
(16) Acetic acid ^b^	1.95 (s)	1.95 (s)
(17) Lactic acid ^b^	4.09 (m), 1.30 (d, J = 10.0 Hz)	4.09 (m), 1.32 (m)
(18) Malonic acid ^b^	3.08 (s)	3.10 (s)
(19) Succinic acid ^b^	2.51 (s)	—
(20) Pimelic acid ^b^	2.10 (d, J = 18.8 Hz), 1.55 (m), 1.30 (m)	2.11 (m), 1.59 (m), 1.30 (m)
(21) Ascorbic acid ^c^	4.47 (d, J = 7.8 Hz), 3.72 (m)	4.56 (d, J = 7.9 Hz), 3.72 (m)
**Secondary Metabolites**		
(22) Stigmasterol ^b^	0.78 (m), 0.82 (m), 0.94 (m), 1.02 (s), 1.06 (d, J = 7.1 Hz), 5.05 (m), 5.16 (m), 5.38 (s)	—
(23) Lupeol ^b^	—	4.55(s), 1.68 (s), 1.07 (s), 0.89 (s), 0.86 (s), 0.82 (s), 0.83 (s), 0.75 (s)
(24) Stigmasterol-*β*-D-glucoside ^b^	—	5.10 (d, J = 3.9 Hz), 4.29 (m), 4.9 (m), 4.01 (m), 2.49 (m), 2.11 (m), 2.03 (m), 1.97 (m), 1.87 (m), 1.83 (m), 1.73 (m), 1.71(m), 1.59 (m), 1.54 (m), 1.43 (m), 1.37 (m), 1.26 (m), 1.12 (m), 1.08 (m), 1.03 (m) 1.02 (m), 1.01 (m), 0.97 (m), 0.95 (m), 0.93 (m), 0.91 (m)
(25) β-Sitosterol ^b^	5.39 (d, J = 3.8 Hz), 1.02 (s), 0.83 (m), 0.78 (m)	—
(26) Clinacoside A ^b^	2.86 (s), 3.81 (m), 4.09 (m), 4.05 (m), 4.70 (d, J = 3.5 Hz), 7.19 (s)	—
(27) Clinacoside B ^b^	6.94 (d, J = 10.7 Hz), 4.25 (m), 4.23 (m), 4.01 (d, J = 8.2 Hz), 3.94 (m), 2.51 (s)	—
(28) Clinacoside C ^b^	4.05 (m), 3.84 (m), 3.74 (m), 2.65 (s)	4.04 (m), 3.83 (m), 3.75 (m), 2.67 (s)
(29) Cycloclinacoside A1 ^b^	4.74 (m), 4.70 (d, J = 3.5 Hz), 3.49 (d, J = 4.9 Hz), 3.47 (m), 4.09 (m), 4.62 (d, J = 16.8 Hz)	—
(30) Cycloclinacoside A2 ^b^	4.70 (d, J = 3.5 Hz), 3.32 (m)	—
(31) Clinamide A ^b^	7.32 (d, J = 5.9 Hz), 6.94 (d, J = 10.7 Hz), 3.62 (d, J = 8.4 Hz), 3.47 (m), 3.08 (s)	—
(32) Clinamide B ^b^	7.69 (m), 6.65 (d, J = 4.1 Hz), 4.17 (m), 3.55 (m), 2.75 (s), 2.08 (s)	—
(33) Orientin ^b^	7.69 (m), 7.47 (m), 7.13 (d), 6.94 (d, J = 10.7 Hz), 6.59 (s), 6.25 (m), 4.09 (m), 3.66 (m), 3.52 (m)	—
(34) Isoorientin ^b^	7.32 (d, J = 5.9 Hz), 6.59 (s), 6.52 (s), 3.52 (m), 3.47 (m)	—
(35) Vitexin ^b^	8 (d, J = 8.9 Hz), 6.26 (m), 4.88−3.76 (m)	—
(36) Isovitexin ^b^	8 (d, J = 8.9 Hz), 6.85 (d, J = 14.8 Hz), 6.84 (s), 6.77(s)	—
(37) Schaftoside ^b^	8 (d, J = 8.9 Hz), 6.94 (d, J = 10.7 Hz), 6.77 (s), 3.95–3.21 (m)	—
(38) Isoschaftoside ^b^	8 (d, J = 8.9 Hz), 6.94 (d, J = 10.7 Hz), 6.65 (d, J = 4.1 Hz), 4.85 (m), 4.07 (dd, J = 18.3,), 3.94 (m), 3.85 (m), 3.75 (m), 3.64 (m), 3.53 (m), 3.47 (m)	—
(39) Epigallocatechin ^b^	7.92 (m), 6.52 (s), 2.70 (m)	—
(40) 3-O-Methylgallic acid ^b^	7.19 (s), 3.85 (s)	—
(41) Catechin ^b^	3.94 (m), 2.84 (m)	—
(42) Gallic acid ^b^	6.98 (s)	6.95 (s)

—: Absence of a particular compound in the extract. Compounds with ‘a’ represented identification that was confirmed using Chenomx database matching. Compounds labeled ‘b’ and ‘c’ were compared to previously reported data in *Clinacanthus nutans* by Khoo et al. [16] and Khoo et al. [17], respectively.

## Data Availability

Not applicable.

## Sample Availability

Samples of the compounds are not available from the authors.

## References

[1] Nakano M., Yatsuhashi H., Bekki S., Takami Y., Tanaka Y., Yoshimaru Y., Honda K., Komorizono Y., Harada M., Shibata M. (2022). Trends in hepatocellular carcinoma incident cases in Japan between 1996 and 2019. Sci. Rep..

[2] Musolino V., Gliozzi M., Bombardelli E., Nucera S., Carresi C., Maiuolo J., Mollace R., Paone S., Bosco F., Scarano F. (2020). The synergistic effect of *Citrus bergamia* and *Cynara cardunculus* extracts on vascular inflammation and oxidative stress in non-alcoholic fatty liver disease. J. Tradit. Complement. Med..

[3] Takaki A., Kawai D., Yamamoto K. (2013). Multiple hits, including oxidative stress, as pathogenesis and treatment target in non-alcoholic steatohepatitis (NASH). Int. J. Mol. Sci..

[4] Masarone M., Rosato V., Dallio M., Gravina A., Aglitti A., Loguercio C., Federico A., Persico M. (2018). Role of oxidative stress in pathophysiology of nonalcoholic fatty liver disease. Oxidative Med. Cell. Longev..

[5] Bagherniya M., Nobili V., Blesso C., Sahebkar A. (2018). Medicinal plants and bioactive natural compounds in the treatment of non-alcoholic fatty liver disease: A clinical review. Pharmacol. Res..

[6] Abu-Serie M., Habashy N. (2020). *Vitis vinifera* polyphenols from seedless black fruit act synergistically to suppress hepatotoxicity by targeting necroptosis and pro-fibrotic mediators. Sci. Rep..

[7] Lin C., Chen H., Lung C., Chen H. (2021). Recent advancement in anticancer activity of *Clinacanthus nutans* (Burm. f.) Lindau. Evid.-Based Complement. Altern. Med..

[8] Azemi A., Mokhtar S., Rasool A. (2020). *Clinacanthus nutans* leaves extract reverts endothelial dysfunction in type 2 diabetes rats by improving protein expression of eNOS. Oxidative Med. Cell. Longev..

[9] Ahmad Azam A., Ismail I., Kumari Y., Shaikh M., Abas F., Shaari K. (2020). The anti-neuroinflammatory effects of *Clinacanthus nutans* leaf extract on metabolism elucidated through ^1^H NMR in correlation with cytokines microarray. PLoS ONE.

[10] Nik Abd Rahman N., Nurliyana M., Afiqah M., Osman M., Hamid M., Lila M. (2019). Antitumor and antioxidant effects of *Clinacanthus nutans* Lindau in 4 T1 tumor-bearing mice. BMC Complement. Altern. Med..

[11] Kong H.S., Abdullah Sani N. (2018). Antimicrobial properties of the acetone leaves and stems extracts of *Clinacanthus nutans* from three different samples/areas against pathogenic microorganisms. Int. Food Res. J..

[12] Khoo L., Audrey Kow S., Lee M., Tan C., Shaari K., Tham C., Abas F. (2018). A comprehensive review on phytochemistry and pharmacological activities of *Clinacanthus nutans* (Burm.f.) Lindau. Evid. Based Complement. Altern. Med..

[13] Yeo B., Yap Y., Koh R., Ng K., Chye S. (2018). Medicinal properties of *Clinacanthus nutans*: A review. Trop. J. Pharm. Res..

[14] Hong J., Yang L., Zhang D., Shi J. (2016). Plant Metabolomics: An indispensable system biology tool for plant science. Int. J. Mol. Sci..

[15] Emwas A., Roy R., McKay R., Tenori L., Saccenti E., Gowda G., Raftery D., Alahmari F., Jaremko L., Jaremko M. (2019). NMR spectroscopy for metabolomics research. Metabolites.

[16] Khoo L., Kow A., Maulidiani M., Ang M., Chew W., Lee M., Tan C.P., Shaari K., Tham C.L., Abas F. (2018). ^1^H-NMR metabolomics for evaluating the protective effect of *Clinacanthus nutans* (Burm. f) Lindau water extract against nitric oxide production in LPS-IFN-γactivated RAW 264.7 macrophages. Phytochem. Anal..

[17] Khoo L., Mediani A., Zolkeflee N., Leong S., Ismail I., Khatib A., Shaari K., Abas F. (2015). Phytochemical diversity of *Clinacanthus nutans* extracts and their bioactivity correlations elucidated by NMR based metabolomics. Phytochem. Lett..

[18] Albuquerque B., Heleno S., Oliveira M., Barros L., Ferreira I. (2021). Phenolic compounds: Current industrial applications, limitations and future challenges. Food Funct..

[19] Kaurinovic B., Vastag D. (2019). Flavonoids and phenolic acids as potential natural antioxidants. Antioxidants.

[20] Minatel I., Borges C., Ferreira M., Gomez H., Chen C., Lima G. (2017). Phenolic Compounds: Functional properties, impact of processing and bioavailability. Phenolic Compd. Biol. Act..

[21] Tan L., Khaw K., Ong Y., Khan T., Lee L., Lee W., Goh B. (2020). An overview of *Clinacanthus nutans* (Burm. f.) Lindau as a medicinal plant with diverse pharmacological values. Plant-Derived Bioactives.

[22] Mustapa A., Martin Á., Mato R., Cocero M. (2015). Extraction of phytocompounds from the medicinal plant *Clinacanthus nutans* Lindau by microwave-assisted extraction and supercritical carbon dioxide extraction. Ind. Crops Prod..

[23] Sarega N., Imam M., Ooi D., Chan K., Md Esa N., Zawawi N., Ismail M. (2016). Phenolic rich extract from *Clinacanthus nutans* attenuates hyperlipidemia-associated oxidative stress in rats. Oxidative Med. Cell. Longev..

[24] Hamid H., Yahya I., Yusoff M., Zareen S. (2016). Bioassay-guided isolation and antioxidant activity of sulfur-containing compounds from *Clinacanthus nutans*. J. Chin. Chem. Soc..

[25] Zhang Q., Lin L., Ye W. (2018). Techniques for extraction and isolation of natural products: A comprehensive review. Chin. Med..

[26] Yang H.S., Peng T.W., Madhavan P., Abdul Shukkoor M., Akowuah G. (2013). Phytochemical analysis and antibacterial activity of methanolic extract of *Clinacanthus nutans* leaf. Int. J. Drug Dev. Res..

[27] Yusof Z., Ramasamy S., Mahmood N., Yaacob J. (2018). Vermicompost supplementation improves the stability of bioactive anthocyanin and phenolic compounds in *Clinacanthus nutans* Lindau. Molecules.

[28] Ahmadinejad F., Geir Møller S., Hashemzadeh-Chaleshtori M., Bidkhori G., Jami M. (2017). Molecular mechanisms behind free radical scavengers’ function against oxidative stress. Antioxidants.

[29] Engwa G. (2018). Free radicals and the role of plant phytochemicals as antioxidants against oxidative stress-related diseases. Phytochem. Source Antioxid. Role Dis. Prev..

[30] Haida Z., Nakasha J., Hakiman M. (2020). In vitro responses of plant growth factors on growth, yield, phenolics content and antioxidant activities of *Clinacanthus nutans* (Sabah Snake Grass). Plants.

[31] Flora S. (2009). Structural, chemical and biological aspects of antioxidants for strategies against metal and metalloid exposure. Oxidative Med. Cell. Longev..

[32] Killian B., Yuan T., Tsai C., Chiu T., Chen Y., Chan C. (2020). Emission-related heavy metal associated with oxidative stress in children: Effect of antioxidant intake. Int. J. Environ. Res. Public Health.

[33] Vajrabhaya L., Korsuwannawong S. (2016). Cytotoxicity evaluation of *Clinacanthus nutans* through dimethylthiazol diphenyltetrazolium bromide and neutral red uptake assays. Eur. J. Dent..

[34] Mohd Roslan S., Zakaria Y., Abdullah H. (2018). Cytotoxicity of *Clinacanthus nutans* and mechanism of action of its active fraction towards human cervical cancer cell line, HeLA. J. Sains Kesihat. Malays..

[35] Liew S., Stanbridge E., Yusoff K., Shafee N. (2012). Hypoxia affects cellular responses to plant extracts. J. Ethnopharmacol..

[36] Kim H., Xue X. (2020). Detection of total reactive oxygen species in adherent cells by 2′,7′-dichlorodihydrofluorescein diacetate staining. J. Vis. Exp..

[37] Zhang J., Wang X., Vikash V., Ye Q., Wu D., Liu Y., Dong W. (2016). ROS and ROS-mediated cellular signaling. Oxidative Med. Cell. Longev..

[38] Kučera O., Endlicher R., Roušar T., Lotková H., Garnol T., Drahota Z., Červinková Z. (2014). The effect of tert-butyl hydroperoxide-induced oxidative stress on lean and steatotic rat hepatocytes in vitro. Oxidative Med. Cell. Longev..

[39] Domanski A., Lapshina E., Zavodnik I. (2005). Oxidative processes induced by tert-butyl hydroperoxide in human red blood cells: Chemiluminescence studies. Biochemistry.

[40] Crane D., Häussinger D., Graf P., Sies H. (1983). Decreased flux through pyruvate dehydrogenase by thiol oxidation during t-butyl hydroperoxide metabolism in perfused rat liver. Hoppe-Seyler’s Z. Für Physiol. Chem..

[41] Radić K., Vinković Vrček I., Pavičić I., Čepo D. (2020). Cellular antioxidant activity of Olive Pomace extracts: Impact of gastrointestinal digestion and cyclodextrin encapsulation. Molecules.

[42] Kim Y., Hwang J., Sung S., Jeon Y., Jeong J., Jeon B., Moon S.-H., Park P.-J. (2015). Antioxidant activity and protective effect of extract of *Celosia cristata L*. flower on tert-butyl hydroperoxide-induced oxidative hepatotoxicity. Food Chem..

[43] Grauzdytė D., Pukalskas A., Viranaicken W., El Kalamouni C., Venskutonis P. (2018). Protective effects of *Phyllanthus phillyreifolius* extracts against hydrogen peroxide induced oxidative stress in HEK293 cells. PLoS ONE.

[44] Martín M., Ramos S., Mateos R., Granado Serrano A., Izquierdo-Pulido M., Bravo L., Goya L. (2008). Protection of human HepG2 cells against oxidative stress by cocoa phenolic extract. J. Agric. Food Chem..

[45] Francenia Santos-Sánchez N., Salas-Coronado R., Villanueva-Cañongo C., Hernández-Carlos B. (2019). Antioxidant compounds and their antioxidant mechanism. Antioxidants.

[46] Alam A., Ferdosh S., Ghafoor K., Hakim A., Juraimi A., Khatib A., Sarker Z. (2016). *Clinacanthus nutans*: A review of the medicinal uses, pharmacology and phytochemistry. Asian Pac. J. Trop. Med..

[47] Peik Lin T. (2020). A minireview on phytochemical and medicinal properties of *Clinacanthus nutans*. J. Appl. Pharm. Sci..

[48] Teshima K., Kaneko T., Ohtani K., Kasai R., Lhieochaiphant S., Picheansoonthon C., Yamasaki K. (1998). Sulfur-containing glucosides from *Clinacanthus nutans*. Phytochemistry.

[49] Gholkar M., Li J., Daswani P., Tetali P., Birdi T. (2021). ^1^H nuclear magnetic resonance-based metabolite profiling of guava leaf extract: An attempt to develop a prototype for standardization of plant extracts. BMC Complement. Med. Ther..

[50] Escudero N., Marhuenda-Egea F., Ibanco-Cañete R., Zavala-Gonzalez E., Lopez-Llorca L. (2014). A metabolomic approach to study the rhizodeposition in the tritrophic interaction: Tomato, *Pochonia chlamydosporia* and *Meloidogyne javanica*. Metabolomics.

[51] Chen M., Wang T., Jiang Z., Shan C., Wang H., Wu M., Wu M.-J., Zhang S., Zhang Y., Zhang L.-Y. (2014). Anti-inflammatory and hepatoprotective effects of total flavonoid C-glycosides from *Abrus mollis* extracts. Chin. J. Nat. Med..

[52] Voynow J., Shinbashi M. (2021). Neutrophil elastase and chronic lung disease. Biomolecules.

[53] Stankovic M., Niciforovic N., Topuzovic M., Solujic S. (2011). Total phenolic content, flavonoid concentrations and antioxidant activity, of the whole plant and Plant Parts Extracts from *Teucrium Montanum* L. Var. *Montanum*, F. *Supinum* (L.) Reichenb. Biotechnol. Biotechnol. Equip..

[54] Shen Q., Zhang B., Xu R., Wang Y., Ding X., Li P. (2010). Antioxidant activity in vitro of the selenium-contained protein from the Se-enriched *Bifidobacterium animalis* 01. Anaerobe.

[55] Lee K., Oh Y., Cho W., Ma J. (2015). Antioxidant and anti-inflammatory activity determination of one hundred kinds of pure chemical compounds using offline and online screening HPLC assay. Evid. Based Complement. Altern. Med..

[56] Chew Y., Goh J., Lim Y. (2009). Assessment of in vitro antioxidant capacity and polyphenolic composition of selected medicinal herbs from Leguminosae family in Peninsular Malaysia. Food Chem..

[57] Benzie I., Strain J. (1999). [2] Ferric reducing/antioxidant power assay: Direct measure of total antioxidant activity of biological fluids and modified version for simultaneous measurement of total antioxidant power and ascorbic acid concentration. Oxid. Antioxid. Part A.

[58] Slamenova D., Kozics K., Hunakova L., Melusova M., Navarova J., Horvathova E. (2013). Comparison of biological processes induced in HepG2 cells by tert-butyl hydroperoxide (t-BHP) and hydroperoxide (H_2_O_2_): The influence of carvacrol. Mutat. Res./Genet. Toxicol. Environ. Mutagenesis.

[59] Wang H., Joseph J. (1999). Quantifying cellular oxidative stress by dichlorofluorescein assay using microplate reader. Free Radic. Biol. Med..

